# Clinical characteristics and potential association to Parkinson’s disease and dementia with Lewy bodies in patients with major depressive disorder who received maintenance electroconvulsive therapy: a retrospective chart review study

**DOI:** 10.1186/s12888-023-04743-7

**Published:** 2023-04-11

**Authors:** Shun Kudo, Takahito Uchida, Hana Nishida, Akihiro Takamiya, Toshiaki Kikuchi, Bun Yamagata, Masaru Mimura, Jinichi Hirano

**Affiliations:** 1 Department of Neuropsychiatry, Keio University School of Medicine, 35 Shinanomachi, Shinjuku-Ku, Tokyo, 160-8582 Japan; 2 Department of Psychiatry, Saitama City Hospital, Saitama, Japan; 3 Department of Psychiatry, Melbourne Neuropsychiatry Centre, The University of Melbourne, Victoria, Australia

**Keywords:** DaT-SPECT, Dementia with Lewy bodies, Major depressive disorder, Maintenance ECT, MIBG scintigraphy, Parkinson’s disease

## Abstract

**Background:**

Maintaining remission after electroconvulsive therapy (ECT) is clinically relevant in patients with depression, and maintenance ECT has been introduced in patients who fail to maintain remission after ECT. However, the clinical characteristics and biological background of patients who receive maintenance ECT are poorly understood. Thus, this study aimed to examine the clinical background of patients who underwent maintenance ECT.

**Methods:**

Patients with major depressive disorder who underwent ECT followed by maintenance ECT (mECT group) and those who did not (acute ECT [aECT] group) were included. Clinical characteristics, including the results of neuroimaging examinations for Parkinson’s disease (PD) and dementia with Levy body (DLB) such as myocardial 123I-metaiodobenzylguanidine (MIBG) scintigraphy and dopamine transporter imaging single-photon emission computerized tomography (DaT-SPECT), were compared between the groups.

**Results:**

In total, 13 and 146 patients were included in the mECT and aECT groups, respectively. Compared to the aECT group, the mECT group showed a significantly higher prevalence of melancholic features (92.3% vs. 27.4%, *p* < 0.001) and catatonic features (46.2% vs. 9.6%, *p* = 0.002). Overall, 8 of the 13 patients in the mECT group and 22 of the 146 patients in the aECT group underwent neuroimaging examinations for PD/DLB. The rate of patients examined is significantly higher in the mECT group than in the aECT group (61.5% vs. 11.2%, *p* < 0.001). Among the groups examined, 7/8 patients in the mECT group and 16/22 patients in the aECT group showed relevant neuroimaging findings for PD/DLB; the positive rate was not significantly different between the two groups (87.5% vs. 72.7%, *p* = 0.638).

**Conclusions:**

Patients who receive acute and maintenance ECT may have underlying neurodegenerative diseases, including PD/DLB. Investigating the neurobiology of patients who receive maintenance ECT is important for developing appropriate treatments for depression.

## Introduction

Depression is a debilitating mental disorder that requires treatment with different modalities [1, 2] and the response rate is only 43–53% and 42–64% with pharmacotherapy and cognitive behavioral therapy, respectively [3–5]. Given the evidence for the lower efficacy of pharmacotherapy in older patients with depression [6], it is important to consider interventions beyond pharmacotherapy for this population. Patients with depression who fail to respond to multiple treatments are considered to have treatment-resistant depression (TRD), and electroconvulsive therapy (ECT) has been used for TRD [1]. ECT is regarded as an effective treatment for TRD, with a remission rate of 50–60% [7, 8]. In addition, a meta-analysis indicated that older age is a predictor for good response to ECT [9], implying that ECT may be a reasonable treatment option for this population. However, previous studies have shown that 40–60% of patients relapse after acute ECT, even with continuous treatment with antidepressants [10–12], and patients do not respond adequately to pharmacotherapy in relapse after ECT [12]. Thus, patients who subsequently relapse after ECT might be regarded as the most severe cases, and maintenance ECT is reported to provide therapeutic benefits for these patients [13].

Maintenance ECT is defined as treatment with ECT once every 1–3 months for at least 6 months [14, 15], and approximately 10–15% of patients with affective disorders require maintenance ECT [16]. Accumulating evidence indicates the efficacy of maintenance ECT for the relapse and recurrence of depression. Patients who received maintenance ECT plus pharmacotherapy after acute ECT had a 54% lower risk of recurrence and relapse at 12 months than did those who received pharmacotherapy alone [13]. Moreover, in older patients with psychotic depression, the 2-year relapse rate was only 8% with nortriptyline plus maintenance ECT and 62% with nortriptyline alone [17]. The American Psychiatric Association guideline for the treatment of major depressive disorder (MDD) also recommends maintenance ECT as a treatment for patients with TRD who fail to maintain remission with pharmacotherapy and psychotherapy and have a history of response to acute ECT [18]. Thus, maintenance ECT is the treatment of choice for patients who have experienced the most difficulty in maintaining remission, and it is not uncommon to encounter such patients in clinical settings. However, limited attention has been paid to the clinical background of patients undergoing maintenance ECT.

Several studies have focused on the association of depression with Parkinson’s disease (PD) and with dementia with Lewy bodies (DLB). A Swedish longitudinal follow-up cohort study reported that the risk of PD was three times higher in patients diagnosed with depression over the age of 50 than in the control group [19]. In addition, recent studies have pointed out that potential factors in older adults with MDD might be due to progressive neurodegenerative disorder or vulnerability [20]. A Japanese study reported that 13.8% of patients diagnosed with depression at age ≥ 50 years were diagnosed with DLB after hospitalization [21]. Moreover, 50% of patients with PD have depressive symptoms [22], and antidepressants are less effective for depression associated with PD [23]. Thus, it has been suggested that depression might be associated with PD and DLB, and depression associated with PD may be linked to treatment resistance [23].

This study aimed to examine the clinical background of patients who underwent maintenance ECT, including neuroimaging markers associated with PD and DLB. Towards this goal, we performed a chart review using a dataset of the ECT cohort that included patients who received maintenance ECT and those who did not, and compared the clinical factors between these patients.

## Materials and methods

### Study design and patients

This study was approved by the ethics committee of Keio University School of Medicine, Tokyo, Japan. The need for written informed consent for this study was waived under the validation of the ethics committee. We further confirm that all methods were employed in accordance with relevant guidelines and regulations. Data were obtained from the Keio Neuropsychiatric ECT database. The database included patients who underwent ECT for treatment of depressive episodes at Keio University Hospital, Tokyo, Japan. In our institution, acute ECT sessions are performed for patients with depression refractory to medications, the need for rapid recovery, and/or according to the patients/relatives’ preference for ECT. Meanwhile, maintenance ECT is provided to patients who fail to maintain remission after acute ECT based on the clinical decision of the psychiatrist in charge.

The current study included patients registered between April 2012 and March 2019. The inclusion criteria were as follows: (1) met the diagnostic criteria for MDD according to the Diagnostic Statistical Manual of Mental Disorders (DSM) IV-TR or DSM-5 [24, 25], as determined by at least two certified psychiatrists and (2) received acute ECT alone or acute ECT followed by maintenance ECT for the treatment of depressive episodes during the study period. The exclusion criteria were those who received acute ECT before the study period and had already been started on maintenance ECT. For patients who received several acute ECTs during the study period, the first acute ECT was selected for our analysis.

Maintenance ECT was defined as treatment with ECT for > 6 months according to the criteria used in the meta-analysis by Elias et al. [13]. The course of ECT other than maintenance ECT was defined as acute ECT. The patients who underwent maintenance ECT were categorized to the mECT group, while the other patients were categorized to the acute ECT (aECT) group.

### ECT procedure

All patients were treated with bitemporal stimulation using a half-age method with a brief-pulse (0.5 ms) square-wave ECT device (Thymatron System IV devices; Somatics, Inc., Lake Bliff, IL, USA) for each ECT session. General anesthesia was induced via intravenous administration of sodium thiopental (3–5 mg/kg), propofol (1 mg/kg), or sevoflurane. Succinylcholine (0.5–1.0 mg/kg) was used to induce muscle relaxation. A two-channel electroencephalogram was monitored to ensure adequate seizure duration. The patients were re-stimulated at a higher intensity (i.e., a 50% increase) when the seizure duration was less than 20 s.

Acute ECT was performed two–three times per week until a stable response was obtained or until no response to acute ECT was confirmed. The number of ECT sessions for each acute ECT was decided by the psychiatrist in charge based on clinical judgement. Maintenance ECT was considered for patients who relapsed within a short period after acute ECT even with adequate antidepressants. The psychiatrist in charge made the decision to introduce maintenance ECT to these patients, and the interval between each maintenance ECT session was decided based on clinical observations. If a patient who had received maintenance ECT developed an acute worsening of their depressive symptoms, the patient underwent acute ECT, and maintenance ECT was re-introduced after symptomatic improvement was achieved.

### Data collection

We extracted clinical information at the time of the first acute ECT for both the mECT and aECT groups, including sex, age at the first ECT, age at MDD onset, number of ECT sessions, number of previous depressive episodes, duration of MDD, duration of the current depressive episode, number of suicidal attempts during the current depressive episode, and family history of MDD. We also extracted the dosage of psychotropics prescribed for included subjects on the day before the first ECT session and converted to a Defined Daily Dose (DDD) [26]. To evaluate cognitive function, we obtained the Mini Mental State Examination (MMSE) score before and after the acute ECT sessions.

Furthermore, the results of neuroimaging markers for PD/DLB, including myocardial 123I-metaiodobenzylguanidine (MIBG) scintigraphy and dopamine transporter imaging single-photon emission computerized tomography (DaT-SPECT), were collected for both the mECT and aECT groups during the study period. These were ordered when psychiatrists or neurologists suspected complications of PD/DLB based on clinical symptoms, including motor symptoms or cognitive symptoms associated with PD or DLB.

Myocardial MIBG scintigraphy and DaT-SPECT have been shown to be helpful in the diagnosis of PD and DLB. DLB Consortium guidelines indicate reduced dopamine transporter uptake in the basal ganglia on SPECT and reduced myocardial MIBG uptake as indicative biomarkers of DLB [27]. In addition, the clinical guideline for PD published by the Movement Disorder Society suggested reduced myocardial MIBG uptake as supportive criteria for PD diagnosis. Further, normal functional neuroimaging of the presynaptic dopaminergic system was indicated as the absolute exclusion criteria for PD [28].

In our institution, myocardial MIBG scintigraphy involves intravenous administration of 111 MBq of 3-iodobenzylguanidine (123I), followed by cardiac scintigraphy using a gamma camera at 15 min and 3–6 h after injection. An early H/M ratio of less than 2.51 or delayed H/M ratio of less than 2.2 is considered positive for myocardial MIBG scintigraphy [29]. In DaT-SPECT, 111–185 MBq of ioflupane (123I) is administered intravenously, and scintigraphy of the head is obtained at 3–6 h intervals. A specific binding ratio of ≤ 4.5 is considered positive for DaT-SPECT [30]. In this study, patients were classified as having a PD/DLB pattern if either the DaT-SPECT or myocardial MIBG scintigraphy results was positive.

Baseline severity of illness was assessed using the Clinical Global Impression–Severity Scale (CGI-S). Briefly, the CGI-S is a 7-point scale, where one means normal or not at all ill, and seven means the most severely ill [31]. In addition, the presence of psychotic, melancholic, and catatonic features, defined based on the criteria of the DSM-IV-TR or DSM-5 at baseline, was also assessed. Treatment response to acute ECT courses was assessed using the clinical Global Impression Improvement scale (c-CGI) [32]. The c-CGI is a 4-point scale where 1 means excellent response and 4 means poor response. This scale has been used in previous retrospective chart reviews of acute ECT. Three board-certified psychiatrists (A.T., T.U., and H.J.) retrospectively assessed CGI-S, presence of psychotic, melancholic, and catatonic features, and c-CGI independently based on the patients’ medical charts, and any disagreements were resolved by discussion.

### Statistical analyses

First, clinical characteristics and symptoms were compared between the mECT and aECT groups. Second, because the comparison of all patients with different observation periods may be qualitatively different, we compared patients who had been followed up in our institute for more than 1 year between the aECT group and the mECT group. Third, the mECT group was divided into patients with and without the PD/DLB pattern, and the differences in clinical characteristics between the groups were investigated. Differences in participant characteristics between groups were examined using an independent t-test for continuous variables and χ2 analysis for categorical variables. All statistical analyses were performed using SPSS version 25.0 (IBM corporation, NY, USA). All analyses were two sided, with an α level of 0.05.

## Results

In total, 159 patients were included in the analysis; of them, 13 and 146 patients were categorized to the mECT and aECT groups, respectively. The intervals between ECT sessions in the mECT group ranged from 2 to 10 weeks. Table 1 summarizes the clinicodemographic characteristics of the patients. The age at onset and age at first ECT were significantly higher in the mECT group. There was no difference in the number of acute ECT sessions between the two groups. Regarding clinical symptoms, the prevalence of melancholic and catatonic features was significantly higher in the mECT group, although the baseline severity was not significantly different between the groups. The treatment response to acute ECT and DDD of psychotropics just before the first ECT session was also not significantly different between the groups. There were also no differences in cognitive function before and after ECT between the two groups.

**Table 1:** Clinical and demographic characteristics of the mECT and aECT groups

	mECT (*n* = 13)	aECT		mECT vs all aECT *p*-value	mECT vs. over 1-year aECT *p*-value
		All (*n* = 146)	Followed up over 1 year (*n* = 73)		
Male sex	3 (23.1)	64 (43.8)	26 (35.6)	0.146	0.529
Age at the first ECT (years)	76.7 [7.4]	62.0 [14.6]	63.5 [13.6]	< 0.001	0.002
Age at MDD onset (years)	64.0[9.1]	55.3 [7.2]	56.5 [16.0]	0.007	0.009
Number of ECT sessions	9.15 [1.62]	8.99 [2.64]	9.32 [2.50]	0.829	0.824
Number of previous depressive episodes	3.5 [1.9]	2.3 [1.8]	2.4 [1.8]	0.016	0.039
Duration of MDD (months)	75.3 [56.7]	76.4 [88.2]	80.8 [96.9]	0.966	0.239
Duration of the current depressive episode (months)	10.1 [9.5]	18.8 [38.2]	18.0 [41.4]	0.414	0.495
Number of suicide attempts	2 (15.4)	20 (13.7)	9 (12.3)	0.564	0.670
Family history of MDD	1 (7.7)	28 (19.2)	16 (21.9)	0.465	0.449
Melancholic features	12 (92.3)	40 (27.4)	15 (20.5)	< 0.001	< 0.001
Psychotic features	3 (23.1)	48 (32.9)	24 (32.9)	0.350	0.747
Catatonic features	6 (46.2)	14 (9.6)	5 (6.8)	0.002	< 0.001
Baseline CGI-S	5.4 [1.3]	4.8 [1.4]	4.8 [1.4]	0.140	0.187
Post-treatment c-CGI	1.4 [0.7]	1.6 [0.9]	1.4 [0.7]	0.475	0.815
**DDD at pre-ECT**					
Antidepressants	0.96 [0.89]	1.24 [0.95]	1.22 [1.01]	0.298	0.381
Antipsychotics	0.19[0.26]	0.29 [0.58]	0.28 [0.49]	0.525	0.492
Anti-anxiety drugs	0.15 [0.21]	0.14 [0.35]	0.18 [0.44]	0.892	0.818
Hypnotics	0.31 [0.43]	0.74 [0.95]	0.71 [0.76]	0.106	0.065
Anti-epileptic drugs	0	18.8 [38.2]	0	0.498	n/a
Mood stabilizers	0	0.02 [0.20]	0	0.764	n/a
Antidementia drugs	0	0.01 [0.06]	0.01 [0.09]	0.683	0.564
**Number of prescriptions at pre-ECT**					
Antidepressants	9 (69.2)	122 (83.6)	62 (84.9)	0.226	0.215
Antipsychotics	7 (53.8)	71 (48.6)	35 (47.9)	0.772	0.728
Anti-anxiety drugs	5 (38.4)	27 (18.5)	14 (19.2)	0.143	0.154
Hypnotics	5 (38.4)	81 (55.5)	45 (61.6)	0.207	0.105
Anti-epileptic drugs	0	6 (4.1)	0	0.451	0.762
Mood stabilizers	0	1 (0.7)	0	0.762	n/a
Antidementia drugs	0	2 (1.4)	2 (2.7)	0.671	0.546
MMSE at pre-ECT ^a^	24.67 [4.51]	27.39 [2.81]	27.21 [3.15]	0.112	0.202
MMSE at post-ECT ^b^	24.00 [2.65]	27.11 [3.10]	27.00 [3.49]	0.097	0.168

Data are present as the mean [SD] or n (%)

^a^mECT, all aECT, and 1 year followed up aECT included 3, 66, and 33 patients, respectively

^b^mECT, all aECT, and 1 year followed up aECT included 3, 46, and 22 patients, respectively

*aECT* Acute electroconvulsive therapy, *cCGI* Clinical Global Impression Improvement scale, *CGI-S* Clinical Global Impression–Severity Scale, *DDD* Defined Daily Dose, *mECT* Maintenance electroconvulsive therapy, *MDD* Major depressive disorder, *MMSE* Mini Mental State Examination

The comparison results between the subgroup of patients in the aECT group who were followed up for more than 1 year after acute ECT (*n* = 73) and the patients in the mECT group are also shown in Table 1. The results also indicated a higher age of onset, higher age of the first ECT, higher prevalence of melancholic and catatonic features in the mECT group, and no significant difference in baseline severity and treatment response to acute ECT between the groups.

**Fig. 1:**
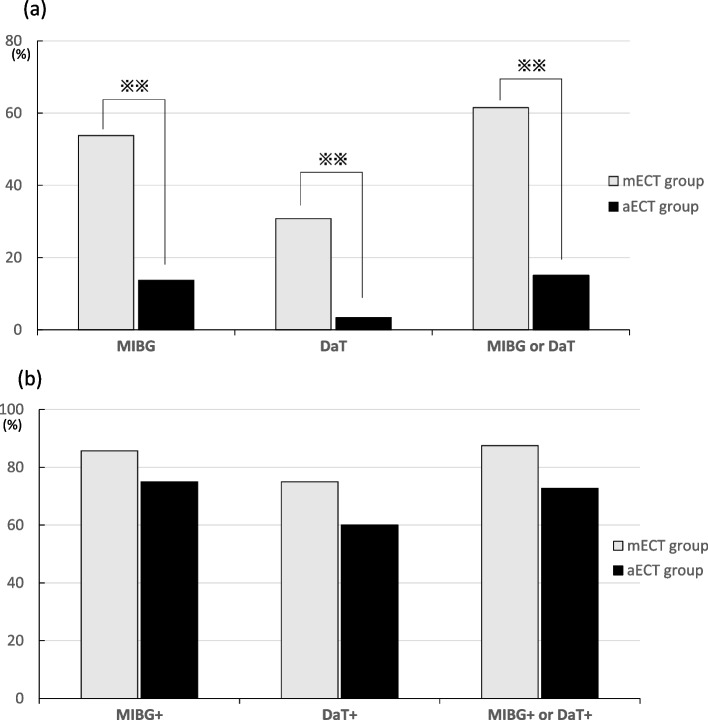
**a** Rate of patients in the mECT and aECT groups who underwent neuroimaging marker testing for PD/DLB. The rate was significantly higher in the mECT group than in the aECT group. **b** Rate of patients with PD/DLB patterns in the mECT and aECT groups. There was no significant difference in the rate between the two groups. Statistical analysis was performed using χ2 analysis for categorical variables. ** *p* < 0.01. MIBG: 123I- metaiodobenzylguanidine scintigraphy, DaT: dopamine transporter imaging single-photon emission computerized tomography

Overall, 8 of the 13 patients (61.5%) in the mECT group underwent examination for neuroimaging markers for PD/DLB for motor symptoms (*n* = 4), cognitive symptoms (*n* = 2), and other reasons (*n* = 2). Meanwhile, 22 of the 146 patients (11.2%) in the aECT group underwent examination for neuroimaging markers for PD/DLB for motor symptoms (*n* = 13), cognitive symptoms (*n* = 5), and other reasons (*n* = 4). The frequency of neuroimaging marker examinations for PD/DLB was significantly higher in the mECT group compared to that in the aECT group (*p* < 0.001) (Fig. 1a). However, the rate of positive results for neuroimaging markers for PD/DLB was not significantly different between the two groups {7/8 (87.5%) in the mECT group vs. 16/22 (72.7%) in the aECT group, *p* = 0.638} (Fig. 1b). There were no significant differences in any characteristics between patients with PD/DLB pattern (*n* = 7) and those without PD/DLB pattern (*n* = 6) in the mECT group.

## Discussion

The clinical characteristics and biological background of patients who receive maintenance ECT are yet to be fully elucidated. The current study showed a higher age of onset, higher age of first ECT, and higher prevalence of melancholic and catatonic features in patients who underwent maintenance ECT than in those who did not. Furthermore, 7 of 13 patients who underwent maintenance ECT showed a PD/DLB pattern, potentially suggesting unique biological characteristics in these patients. However, the positive rate of neuroimaging markers for PD/DLB in the mECT group was not significantly different from that of the aECT group. To the best of our knowledge, this is the first study to focus on the clinical characteristics, including the results of neuroimaging markers for PD/DLB, in patients who underwent maintenance ECT.

The clinical characteristics of the mECT group compared to the aECT group in this study were consistent with the clinical characteristics reported of TRD compared to the non-TRD group [33–35]. That is, clinical characteristics, including old age of onset and melancholic and catatonic features, may be indicators of treatment resistance at all stages of depression and may provide a rationale for the choice of therapies other than conventional antidepressant therapy for depression patients with these features.

The finding that approximately 40% of patients in the mECT group and 15% of patients in the aECT group showed a positive rate of PD/DLB pattern highlights the potential biological background of these patients. Although the rate of positive PD/DLB patterns was not significantly different between the mECT and aECT groups, a significantly higher proportion of patients in the mECT group underwent examination for neuroimaging markers. These findings suggested that a greater proportion of patients in the mECT group had underlying neurodegeneration. In a prospective cohort study [19], 1.1% of patients with depression had comorbidity with PD, and this rate is much lower than that reported in the present study. The high proportion of PD/DLB patterns in the current study suggest that PD/DLB pathology may be involved in the context of treatment resistance in depression. Indeed, depression associated with PD has been reported to respond inadequately to conventional antidepressants, and dopamine dysfunction may be implicated in treatment resistance [23, 36]. Meanwhile, ECT has been reported to be effective for depression associated with PD [37]. Animal studies show that ECT increases D1 and D3 binding in the striatum [38] and increases D1 receptor binding and vesicular monoamine transporter type 2 binding [39]. Thus, ECT acts on the dopaminergic system and may be an effective treatment for depression in the context of dopaminergic abnormalities.

The biological changes that occur in patients undergoing maintenance ECT is still unclear. Our finding that 40% of the patients in the maintenance ECT group had a PD pattern suggests that the development of PD pathology may be related to treatment resistance. Whether the manifestation of PD is caused by maintenance ECT or whether maintenance ECT is only used in the severely depressed group is a topic beyond the focus of this study; however, the various biological effects of maintenance ECT in severely depressed patients should be continued to be examined.

Our study had the following limitations. First, because we did not systematically assess symptoms associated with PD/DLB for all included subjects, we could not diagnose PD or DLB as a comorbidity, even with positive results for neuroimaging markers for PD/DLB. Second, the mECT group could receive more frequent medical assessments than could the aECT group and therefore had a greater probability of receiving neuroimaging markers for PD/DLB, which may have contributed to the higher rate of examination in the mECT group. Third, due to the small sample size, some factors associated with the mECT group may have been overlooked due to type 2 error. Further studies with a larger sample size and a systematic assessment of PD and DLB are warranted to investigate the pathophysiology of treatment-resistant depression.

## Conclusion

The current study found a higher age at onset, higher age at the first ECT, and higher prevalence of melancholic and catatonic features in patients who underwent maintenance ECT. Furthermore, the results of neuroimaging markers for PD/DLB implied that patients who underwent acute and maintenance ECT have impaired dopamine function or postganglionic cardiac autonomic denervation, which are associated with PD/DLB. Investigating the neurobiology of patients who receive maintenance ECT is important for the development of appropriate treatments for depression.

## Data Availability

The datasets used and/or analyzed during the current study are not publicly available in accordance with the provisions of the ethics committee but are available from the corresponding author upon reasonable request.

## Abbreviations

TRD: Treatment-Resistant Depression
ECT: Electroconvulsive Therapy
MDD: Major Depressive Disorder
PD: Parkinson’s disease
DLB: Dementia with Lewy Bodies
DSM: Diagnostic Statistical Manual of Mental Disorders
MIBG: 123I-Metaiodobenzylguanidine
DaT-SPECT: Dopamine Transporter Imaging Single-Photon Emission Computerized Tomography
CGI-S: Clinical Global Impression–Severity Scale
c-CGI: Clinical Global Impression Improvement scale
